# Late-onset renal variant Fabry disease with R112H mutation and mild increase in plasma globotriaosylsphingosine: a case report

**DOI:** 10.3389/fmed.2024.1383309

**Published:** 2024-06-06

**Authors:** Keiko Tanaka, Hitoshi Sugiyama, Hiroshi Morinaga, Akifumi Onishi, Katsuyuki Tanabe, Haruhito A. Uchida, Hiroki Maruyama, Jun Wada

**Affiliations:** ^1^Faculty of Medicine, Dentistry and Pharmaceutical Sciences, Department of Nephrology, Rheumatology, Endocrinology and Metabolism, Okayama University, Okayama, Japan; ^2^Department of Medicine, Kawasaki Medical School General Medical Center and Department of Medical Care Work, Kawasaki College of Health Professions, Okayama, Japan; ^3^Department of Nephrology, Fukuyama City Hospital, Hiroshima, Japan; ^4^Department of Clinical Nephroscience, Niigata University Graduate School of Medical and Dental Sciences, Niigata, Japan; ^5^Niigata Seiro Hospital, Niigata, Japan

**Keywords:** Fabry disease, R112H mutation, migalastat, proteinuria, chronic kidney disease

## Abstract

Fabry disease (FD) is an X-linked disorder resulting in a deficiency of α-galactosidase A (GLA) activity. The R112H mutation of GLA is relatively common in Japanese FD patients, characterized by a late-onset phenotype, almost normal to mild lyso-Gb3 elevation, and mild clinical symptoms, despite low GLA activity. This is due to the structural features of the R112H GLA protein. We herein report the case of a 42-year-old male patient with late-onset FD with a R112H mutation. The patient exhibited only renal involvement with no other organ damage and was successfully treated with galactosidase beta and subsequent migalastat for approximately 10 years. Especially, migalastat was clinically effective in normalizing plasma lyso-Gb3 levels and inhibiting the progression of renal damage associated with FD. Therefore, the use of migalastat in the FD patients with R112H mutation is highly recommended based on this case report.

## Introduction

Fabry disease (FD) is an X-linked recessive disorder caused by *GLA* mutations that result in deficient lysosomal α-galactosidase A (GLA) activity (1). Enzymatic defects lead to systemic lysosomal accumulation of glycolipids, including globotriaosylceramide (Gb3) and globotriaosylsphingosine (lyso-Gb3) (2). Plasma lyso-Gb3 levels are correlated with the severity of FD (3). FD can be classified into the classical type (no residual enzymatic activity and markedly elevated lyso-Gb3 levels) or the late-onset type (low residual enzymatic activity and mildly elevated lyso-Gb3 levels). Affected men with the classical type show angiokeratoma, acroparasthesia, hypohidrosis, and gastrointestinal disorders during childhood or adolescence. In adults, disease progression leads to cerebrovascular disease, cardiac damage, chronic kidney disease, and premature death (4). Men with the late-onset type develop renal and/or cardiac disorders in adulthood, without childhood symptoms. Heterozygous women can manifest both classical and late-onset type of FD owing to random X-chromosomal inactivation (2).

R112H (*c.335G > A*) and M296I (*c.888G > A*) are common *GLA* variants in Japanese patients with FD. R112H has been detected in various countries, whereas M296I is unique to Japanese patients with FD. Among FD patients, R112H and M296I are characterized by a late-onset phenotype, almost normal to mild lyso-Gb3 elevation, and mild clinical symptoms, despite relatively low GLA activity (5, 6). Therefore, the extent of FD involvement in clinical manifestations should be confirmed not only by GLA activity but also by plasma lyso-Gb3 levels, histological Gb3 accumulation, and genotypic characterization. In this paper, we report a case of late-onset renal type FD with R112H mutation, which was successfully treated with galactosidase beta and subsequent migalastat for approximately 10 years. We discuss the characteristics of patients with R112H-mutated FD.

Furthermore, available evidence indicates that migalastat is a suitable treatment option for FD in patients with amenable mutations. Experience has shown its potential to improve quality of life, control gastrointestinal symptoms, stabilize the renal function, and reduce cardiac hypertrophy. Given its efficacy, broad tissue penetration, simple oral regimen, and the limited treatment options that are currently available, migalastat can be considered as first-line therapy in patients receiving enzyme-replacement therapy who experience side effects, have poor compliance with chronic intravenous administration, or in patients with an unstable disease status (7). R112H is an amenable mutation, and we herein report the clinical efficacy of migalastat in an FD patient with R112H.

## Case presentation

A 42-year-old Japanese man was referred to our department with proteinuria (0.6 g/gCr) and mild renal dysfunction (serum creatinine: 1.2 mg/dL). Proteinuria had been noted at a medical checkup conducted when he was 32 of age, but he had not been examined closely. The patient had no medical history other than proteinuria. The patient was a karate instructor and truck driver (height, 167 cm; weight, 77 kg; body mass index, 27.5). He had smoked 15 cigarettes per day for 10 years since his 20s. His clinical laboratory findings showed no immunoglobulin or complement abnormalities. Mulberry bodies were not detected in his urinary sediments.

**Figure 1:**
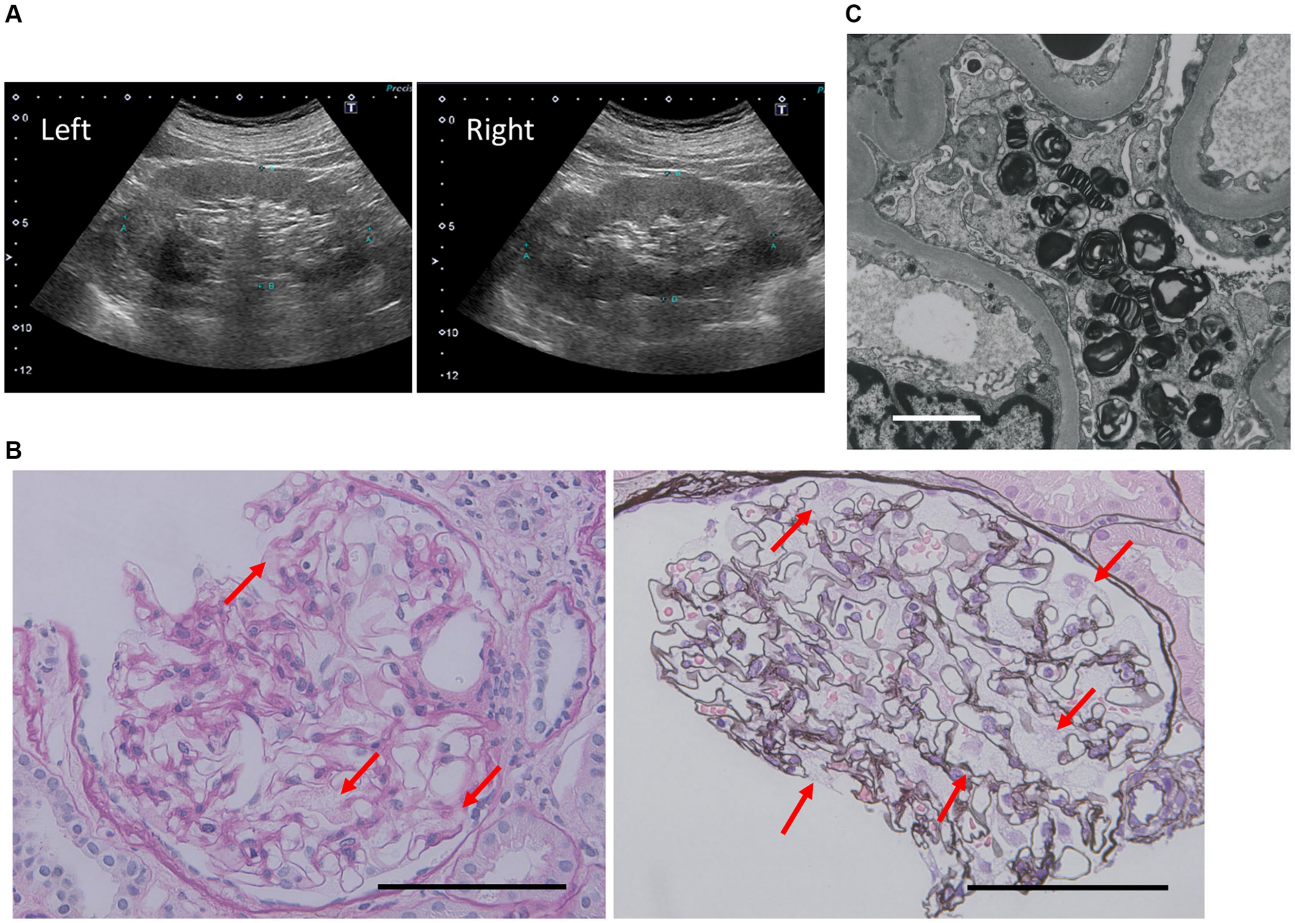
The renal findings. **(A)** On abdominal ultrasonography, both kidneys were of normal size and had normal blood flow, with mild atherosclerotic changes. **(B)** Light microscopy images of the kidney section (periodic acid−schiff staining and periodic acid-methenamine-silver staining) showing foamy podocytes with many vacuolations (arrows). Scale bar: 100 μm. **(C)** Transmission electron microscopy images showing zebra bodies in podocytes. Scale bar: 2 μm.

Abdominal ultrasonography revealed mild arteriosclerotic changes, but no renal or urinary malformations (Figure 1A). A renal biopsy was performed to investigate underlying cause of proteinuria and mild renal dysfunction. Light microscopy of the kidney specimens showed that the glomeruli had swollen podocytes with significant vacuolation (Figure 1B). Approximately half of the glomeruli showed global or segmental sclerosis, and arteriosclerosis was observed in the vessels. Transmission electron microscopy revealed numerous zebra bodies in podocytes (Figure 1C), a characteristic finding of FD.

Reduced GLA activity in leukocyte and increased lyso-Gb3 levels in plasma confirmed the diagnosis of FD (Table 1, Case 1). The plasma Gb3 level was 2.7 μg/mL. A *GLA* mutation (c.335G > A, p.R112H) was identified. Electrocardiography revealed sinus rhythm at a rate of 56 bpm. Echocardiography revealed no cardiac hypertrophy [interventricular septum (IVS) thickness, 9 mm; left ventricular mass index (LVMI), 88 g/m^2^] and no valvular disease, with a normal systolic function (ejection fraction: 62%). He had no acroparesthesia, hypohidrosis, angiokeratoma, or corneal opacity. He also had no symptoms of central nervous system (e.g., dizziness or hearing loss).

**Table 1:** The characteristics of FD patients with R112H mutation previously reported.

Case	Age	Gender	GLA activity	Standard	Plasma lyso-Gb3 levels	Standard	Kidney	Heart	Symptoms	References
1	42	M	(Leukocyte) 1.0 nmol/mgP/h	49.8–116.4	4.1 ng/mL	<2.0	Proteinuria, mild renal dysfunction	None	None	The present case
2	28	F	ー		1 ng/mL		Proteinuria	None	Mild pain	(8)
3	59	M	ー		2 ng/mL		None	None	Mild pain	(8)
4	63	M	ー		2.4 ng/mL		Proteinuria	None	None	(8)
5	13	M	(Leukocyte) 0.2 nmol/mgP/h	20–80	5.0 nmol/L	<2.0	Proteinuria, mild renal dysfunction (Oligonephropathy)	None	None	(9)
6	21	M	(Leukocyte) <1.0 nmol/mgP/h	17–65	5.3 nmol/L	0.38–0.70	Proteinuria, mild renal dysfunction	None	None	(10)
7	61	M	(Serum) 2.7 nmol/h/mL	4≤	(serum) 3.6 ng/mL	<2.0	End-stage renal failure	LVH, IHD, As	Unknown	(11)

M, Male; F, Female; LVH, Left ventricular hypertrophy; IHD, Ischemic heart disease; and As, Mild aortic stenosis.

Agalsidase beta (1 mg/kg, every 2 weeks) was initiated. He also quit smoking. During the 5 years of treatment with agalsidase beta, he exhibited decreased plasma Gb3 (below the detection limit) and a preserved renal function (serum creatinine: 1.3 mg/dL). The patient was then switched to migalastat treatment to reduce hospital visits. Currently, after 4 years of migalastat treatment, his renal findings have not markedly worsened. Angiotensin II receptor blocker (ARB) treatment was initiated because the patient had proteinuria sometimes exceeding 1 g/gCr along with hypertension, and mild changes in serum creatinine (1.4 mg/dL). No findings were suggestive of cardiac involvement (IVS, 10 mm; LVMI, 112 g/m^2^). The patient’s plasma lyso-Gb3 level decreased (1.5 ng/mL).

Intrafamily screening revealed that his 65-year-old mother was heterozygous for the R112H mutation but had no symptoms. Her 90-year-old mother had not undergone genetic testing but had been undergoing hemodialysis since 87 years of age. The patient’s 37-year-old brother and 34-year-old sister had no genetic mutations.

## Discussion

In this presentation, the FD patient with the R112H mutation had a late-onset phenotype, mildly elevated plasma lyso-Gb3 levels, and only mild renal involvement. Migalastat treatment following agalsidase beta treatment successfully prevented marked progression of organ damage for approximately 10 years.

R112H is a relatively common pathogenic variant in Japanese FD patients. Sakuraba reported that in 207 Japanese FD patients, M296I was the most common mutation (allele frequency: 5.8%, 12/207), c.639 + 919G > A and R227* were the second most common mutations (4.3%, 9/207), and R112H, R112C, and R301Q were the third most common mutations (3.9%, 8/207) (6). All five male patients with R112H had the late-onset type. In another study of 236 FD patients from 143 families (12), M296I was the most common (3.5%, 5/143) and R112C was the second most common (2.8%, 4/143), and R112H was the third most common (2.1%, 3/143) as well as the other seven *GLA* variants. Two patients with R112H in the study had renal manifestations (details not available). Furthermore, R112H has been detected in various countries, including Argentina (13), the Czech Republic (14), Austria (15), and Turkey (16), where R112H has been detected in screening tests for patients undergoing hemodialysis or peritoneal dialysis. Thus, R112H has been reported to have a late onset and a tendency to develop renal manifestations. These characteristics are consistent with those observed in the present case.

Table 1 shows the cases of FD patients with R112H whose characteristics are evident in previous reports (8–11). Interestingly, in the R112H mutation, plasma lyso-Gb3 levels were almost normal or only mildly elevated, even though GLA activity was almost as low as in classical mutations. Similar findings were reported by Rombach et al. (17) and Tsukimura et al. (5). All patients, with the exception of case 7, showed only renal involvement. In case 7, it is unclear and controversial whether cardiac manifestations and renal failure requiring hemodialysis could be explained solely by FD. As FD patients age, they are more likely to develop cardiac disorders and progress renal dysfunction from causes other than FD, such as hypertension and atherosclerosis (18). As mentioned above, several cases of renal failure requiring dialysis have been reported in FD patients with R112H, which may have a broad phenotypic spectrum from mild to severe. However, it must be carefully determined whether renal failure requiring dialysis is directly associated with FD severity. In general, FD patients with R112H have mildly elevated plasma lyso-Gb3 levels and mild clinical symptoms.

Tsukimura reported that FD patients with R112H had low GLA activity but substantial amounts of GLA protein, resulting in nearly normal plasma lyso-Gb3 levels (5). R112H is a missense mutation that results in a different amino acid substitution on GLA. R112 is located on the loop comprising the barrel domain of the GLA structure, close to the surface of the molecule, and the amino acid substitution has no effect on the active site (5). This conformational change is thought to affect the stability of the GLA protein, causing partial degradation and denaturation of the mutant GLA protein. The denatured enzyme exhibits slight residual activity, presumably promoting the degradation of lyso-Gb3 in plasma (5). Thus, FD patients with R112H exhibited residual GLA activity, and their plasma lyso-Gb3 levels were lower than those with the other late-onset mutations.

It makes sense to use migalastat, an oral pharmacological chaperone, in patients with the R112H mutation. This is because migalastat would correct the conformational changes in the abundant denatured GLA proteins and greatly increase GLA activity in the R112H mutation. Migalastat has been shown to be responsive to increasing the GLA activity from 2.6 to 14.8% (a 6.7-fold increase) in a GLP-HEK assay of R112H (19). However, the efficacy of migalastat *in vitro* and *in vivo* does not always coincide, and the clinical efficacy of migalastat is only observed in some of the genotypes that meet the criteria for amenable mutations. In patients undergoing migalastat treatment, changes in Lyso-Gb3 levels are not always correlated with increased enzyme activity in leukocytes and Lyso-Gb3 levels may not be correlated with clinical symptoms. As there is no appropriate biomarker for monitoring migalastat treatment, the treating clinician should carefully monitor clinical and laboratory features to confirm the clinical response (20, 21). In this case, migalastat was clinically effective in normalizing plasma lyso-Gb3 levels and inhibiting the progression of renal damage associated with FD. The present case showed a clinical course of renal damage due to atherosclerosis and a better renal prognosis than case 6, which had been untreated for 30 years despite similar GLA activity and lyso-Gb3 levels in plasma (In case 6, serum creatinine was 4–5 mg/dL at age 50). The long-term effects of migalastat are not known, and careful follow-up is needed in the future. Since the latest expert consensus for FD patients states that renoprotective therapies such as ARB and Sodium-glucose transport protein 2 (SGLT2) inhibitors may be effective (22), administration of SGLT2 inhibitors may also be considered in this case.

In conclusion, we reported the case of a patient with a late-onset renal variant of FD with a R112H mutation that responded effectively to migalastat treatment following agalsidase beta treatment. We discussed the pathologic significance of the R112H mutation in FD and the efficacy of migalastat. Therefore, it is important to fully characterize the genotype when considering the clinical course and treatment of FD.

## Data availability statement

The original contributions presented in the study are included in the article/supplementary material; further inquiries can be directed to the corresponding authors.

## Ethics statement

Written informed consent was obtained from the individual(s) for the publication of any potentially identifiable images or data included in this article.

## Author contributions

KeT: Writing – original draft. HS: Writing – review & editing. HMo: Writing – review & editing. AO: Writing – review & editing. KaT: Writing – review & editing. HU: Writing – review & editing. HMa: Writing – review & editing. JW: Writing – review & editing.
